# Coordinated Targeting of the EGFR Signaling Axis by MicroRNA-27a*

**DOI:** 10.18632/oncotarget.1239

**Published:** 2013-08-06

**Authors:** Xiaoli Wu, Mihir K. Bhayani, Cristina T. Dodge, Milena S. Nicoloso, Yunyun Chen, Xiaofeng Yan, Makoto Adachi, Ligy Thomas, Chad E. Galer, Tilahun Jiffar, Curtis R. Pickering, Michael E. Kupferman, Jeffrey N. Myers, George A. Calin, Stephen Y. Lai

**Affiliations:** ^1^ Department of Head and Neck Surgery, University of Texas M.D. Anderson Cancer Center, Houston, TX, USA; ^2^ Division of Gastroenterology, Tongji Hospital of Tongji Medical College, Huazhong University of Science and Technology, Wuhan, Hubei Province, China; ^3^ Department of Experimental Therapeutics, University of Texas M.D. Anderson Cancer Center, Houston, TX, USA; ^4^ Department of Otolaryngology, University of Indiana School of Medicine, Indianapolis, IN, USA; ^5^ Department of Molecular and Cellular Oncology, University of Texas M.D. Anderson Cancer Center, Houston, TX, USA

**Keywords:** miRNA, EGFR, AKT1, mTOR, miRNA-27a*, miRNA-27a-5p

## Abstract

Epidermal growth factor receptor (EGFR) has been characterized as a critical factor in the development and progression of multiple solid tumors, including head and neck squamous cell carcinoma (HNSCC). However, monotherapy with EGFR-specific agents has not been as dramatic as preclinical studies have suggested. Since complex regulation of the EGFR signaling axis might confound current attempts to inhibit EGFR directly, we searched for microRNAs (miRNAs) that may target the EGFR signaling axis. We identified miR-27a (miR-27a-3p) and its complementary or star (*) strand, miR-27a* (miR-27a-5p), as novel miRNAs targeting EGFR, which were significantly downregulated in multiple HNSCC cell lines. Analysis of human specimens demonstrated that miR-27a* is significantly underexpressed in HNSCC as compared to normal mucosa. Increased expression of miR-27a* in HNSCC produced a profound cytotoxic effect not seen with miR-27a. Analysis for potential targets of miR-27a* led to the identification of AKT1 (protein kinase B) and mTOR (mammalian target of rapamycin) within the EGFR signaling axis. Treatment with miR-27a* led to coordinated downregulation of EGFR, AKT1 and mTOR. Overexpression of EGFR signaling pathway components decreased the overall effect of miR-27a* on HNSCC cell viability. Constitutive and inducible expression of miR-27a* in a murine orthotopic xenograft model of oral cavity cancer led to decreased tumor growth. Direct intratumoral injection of miR-27a* inhibited tumor growth *in vivo*. These findings identify miR-27a* as a functional star sequence that exhibits novel coordinated regulation of the EGFR pathway in solid tumors and potentially represents a novel therapeutic option.

## INTRODUCTION

Head and neck squamous cell carcinoma (HNSCC) is the 6^th^ most common cancer worldwide, with approximately 650,000 new cases diagnosed annually [1, 2]. In the United States, HNSCC accounted for 49,260 new cancer cases and 11,480 deaths in 2010 [2]. Despite intensive research and improvements in multimodality treatment, including surgery, radiation and chemotherapy, five-year survival remains at 50-60% [3].

The epidermal growth factor receptor (EGFR) is overexpressed in 40-70% of HNSCC and associated with decreased survival [4, 5]. EGFR has also been characterized as a critical factor in the development and progression of other solid tumor types involving the breast [6], pancreas [7], prostate [8], and uterus [9]. The introduction of a monoclonal antibody to EGFR, cetuximab, has been a significant advance in the treatment of HNSCC patients [10]. However, treatment benefits with cetuximab are seen primarily in combination with radiation therapy, as monotherapy has resulted in less than 10% disease response for recurrent/metastatic HNSCC [11-13]. Additionally, treatment response has not been correlated with alterations in EGFR expression and signaling activity within the tumor [14-16]. Despite extensive biological rationale for the use of EGFR inhibitors, marginal clinical benefits have been observed in broad patient populations [17, 18]. This discordance suggests resistance pathways are present that abrogate the effects of EGFR-specific treatment and alternative approaches to inhibiting EGFR and its downstream mediators may be more effective.

An attractive alternative to small molecule inhibitors and monoclonal antibodies are microRNAs (miRNAs). miRNAs are short, endogenous, non-coding RNA molecules (19-22 nucleotides) that bind to target mRNAs causing RNA interference through translational repression or mRNA degradation [19, 20]. Many miRNAs are located within regions of the human genome which are amplified, deleted or rearranged in cancer [21] and may act as oncogenes and tumor suppressors [22]. MiRNAs have been previously shown to interact with EGFR as both tumor suppressors [23, 24] and oncogenes [25, 26].

Given the current status of EGFR-targeted therapies and the continuing need for effective treatment options, we directly assessed *EGFR* for candidate binding sites of novel miRNAs. This report reveals the tumor suppressive properties of miR-27a* (miR-27a-5p) acting upon the EGFR signaling axis at three independent points, resulting in decreased tumorigenicity *in vitro* and *in vivo*.

## RESULTS

### Identification of miRNA Sequences With Putative Binding Sites in the EGFR mRNA

**Figure 1:**
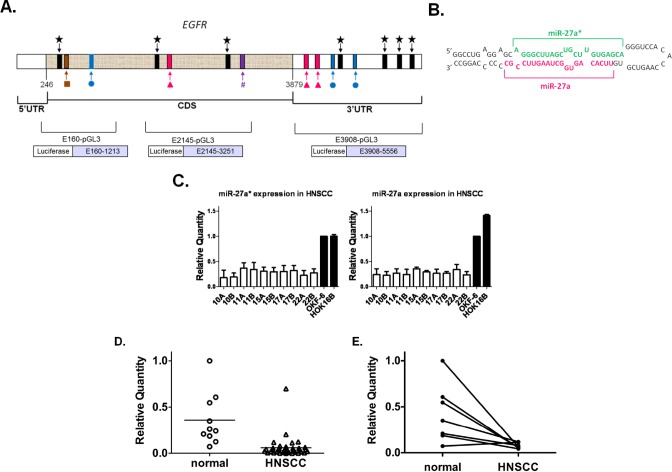
miR-27a* has putative binding sites in EGFR mRNA and shows decreased expression in HNSCC cell lines and human tumor tissues (A) Identification of specific miR-27a(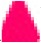), −27a*(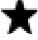), −27b(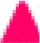), −27b*(#), −7(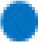), −128(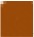) candidate binding sites within EGFR mRNA using *in silico* screening methods; (B) Hairpin representation of the pre-miR-27a with the sequences of miR-27a* (green) and miR-27a (magenta) highlighted; (C) Decreased expression of mature miR-27a and −27a* by qRT-PCR in 10 HNSCC cell lines and normal oral keratinocytes (OKF-6 and HOK16B). Values normalized to OKF-6, p<0.005; (D) Analysis of miR-27a* RNA in human HNSCC and normal mucosal specimens by qRT-PCR revealed an overall decrease in miR-27a* expression levels in HNSCC, p<0.0001; (E) Comparison of miR-27a* levels in matched normal/HNSCC tissue pairs demonstrated decreased expression in the tumors as compared to matched normal tissue, p<0.01.

We used software prediction programs to identify candidate binding sites of miRNAs within the *EGFR* gene and identified miR-7, −27a (miR-27a-3p), −27a*, −27b (miR-27b-3p), −27b* (miR-27b-5p), and −128 (Fig. 1A). Only RNA22 predicted binding sites for the star sequences (miRNA*), which have been generally regarded as non-functional products of miRNA duplex precursor processing (Fig. 1B) [27]. Four potential binding sites were identified for miR-27a* in the 3'-untranslated region (3'UTR) and three in the coding DNA sequence (CDS). MiR-27a, −27b and −7 were predicted to have two target sites in the 3'UTR and one in the CDS. Since miR-27a and 27b had the same seed region sequence, their target sites were the same. Only one target site was identified for miR-27b* or −128 in the CDS.

### miR-27a* and miR-27a Are Downregulated in HNSCC Cells and Tissues

We examined the expression of the candidate miRNAs in HNSCC cell lines by quantitative real-time polymerase chain reaction (qRT-PCR) and found that only miR-27a and −27a* had significantly decreased expression as compared to normal oral keratinocyte cell lines (Fig. 1C). Downregulation of miR-7 was previously reported in glioblastoma [24] along with underexpression of miR-128 in lung cancer [23], but in HNSCC cell lines the expression of these miRNAs was variable as compared to controls (Fig. S1A).

We confirmed these findings by evaluating the expression of miR-27a* in patients with HNSCC. Oral cavity tumors were selected to avoid heterogeneity due to differences in tumor biology at discrete subsites in the head and neck, such as oropharyngeal tumors related to human papilloma virus (HPV) [1]. Analysis of this cohort demonstrated a consistent pattern of significantly decreased miR-27a* expression in HNSCC tumor specimens compared to normal tissues (Fig. 1D). Evaluation of matched normal and tumor samples (n=7 pairs) also showed significantly decreased miR-27a* expression in the tumors (Fig. 1E). We also observed a trend toward decreased miR-27a* expression with advanced tumor stage (p=0.056). This analysis suggested that miR-27a* levels may be significantly altered in HNSCC and perhaps related to prognostic clinical features. Although miR-27a demonstrated decreased expression in HNSCC as compared to normal tissues, analysis of the matched samples did not demonstrate significantly decreased expression in the tumors (Fig. S1B). Evaluation of miR-7 expression did not demonstrate a significant difference in the entire cohort or the matched samples. Based on these findings, we hypothesized that miR-27a* and/or −27a suppression might contribute to the malignant phenotype and that re-introduction of these miRNAs into HNSCC cells might alter tumor behavior.

### miR-27a* Decreases Cell Viability in HNSCC and Other Solid Tumors

**Figure 2:**
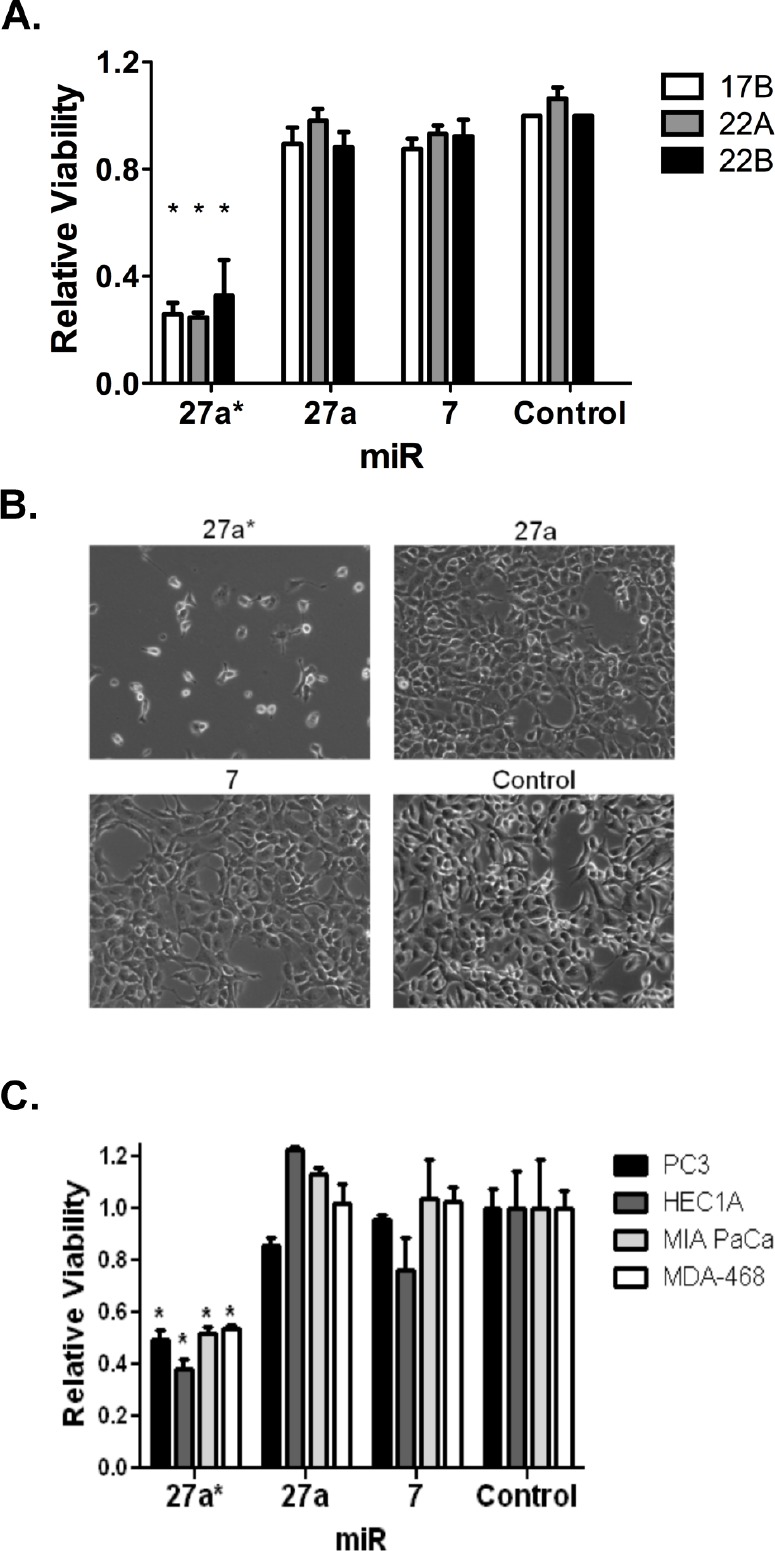
miR-27a* transfection decreases cell viability in HNSCC cells and other solid tumor types (A) Cell viability is decreased at in three HNSCC cell lines after transfection of miR-27a* compared to miR-27a, −7, and -Control, *p<0.001; (B) Photomicrograph details cell number 72 hrs after transfection of miR-27a* mimic into wells containing 20,000 cells each; (C) Cell viability is decreased in other solid tumor cell lines (PC3 prostate, HEC1A endometrial, MIA PaCa pancreas, and MDA-468 breast) after transfection of miR-27a* compared to miR-27a, −7, and -Control, *p<0.001.

To assess the effects of the miRNAs on tumor viability, three HNSCC cell lines were transfected with miR-7, −27a and −27a* mimics. After 72 hr incubation, MTT assays showed a significant decrease in cell viability following miR-27a* transfection but no effect with miR-27a and −7 (Figs. 2A and 2B). The alamarBlue® assay confirmed these results (Fig. S2A). To verify that the effects on cell viability were specific to miR-27a*, we transfected the miR-27a stem-loop structure (miR-27a-SL; Fig. 1B), which is processed into miR-27a and miR-27a*, in HNSCC and oral keratinocyte cells (Fig. S2B). Although there was a decrease in cell viability in HNSCC cells with miR-27a-SL, the effect was not as dramatic as with miR-27a* alone and there was no significant effect of miR-27a*, −27a or miR-27a-SL on HOK16B cells. Additionally, we employed an antagomir, miR-27a*-inhibitor (miR-27a*-IH), to assess potential loss-of-function effects [28]. Cell viability in the HNSCC or HOK16B cells was not affected by miR-27a*-IH (Fig. S2C). We then transfected the miRNA mimics into pancreatic, breast, prostate, and endometrial carcinoma cell lines and observed decreased cell viability with miR-27a*, but not miR-27a and −7 (Fig. 2C), suggesting the biologic effects of miR-27a* are not restricted to HNSCC.

### miR-27a* Inhibits Expression of Multiple EGFR Signaling Axis Components

**Figure 3:**
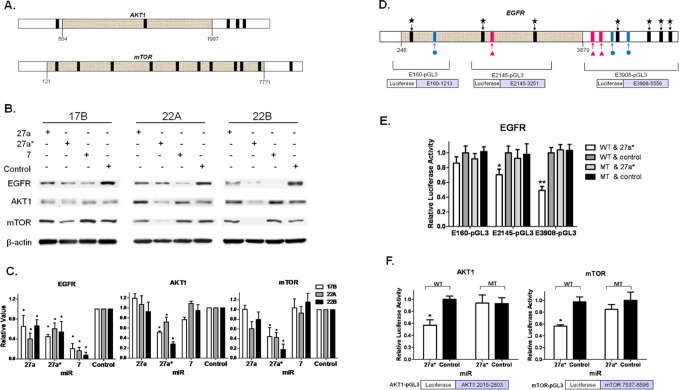
miR-27a* coordinately downregulates the EGFR signaling axis via independent direct interactions with EGFR, AKT1, and mTOR (A) Further *in silico* screening of downstream members of the EGFR signaling axis, identified 5 putative binding sites for miR-27a* (black bars) on AKT1 and 11 binding sites for miR-27a* on mTOR; (B) Immunoblot shows decreased EGFR expression after the transfection of miR-27a*, −27a and −7 precursors. Downstream AKT1 and mTOR are also decreased when transfected with miR-27a*, but not miR-27a or −7; (C) Densitometry analysis quantifies the differences observed in the immunoblots, *p<0.01; (D) Detailed map of reporter plasmid constructs depicting the EGFR sequence placed downstream of luciferase in pGL3; (E) Luciferase assay after transfection of EGFR reporter plasmids and miR-27a* in HNSCC cells demonstrates E2145-pGL3 and E3908-pGL3 have functional binding sites for miR-27a* and that E160-pGL3 is a non-functional site, *p<0.05 and **p<0.005; (F) Luciferase assay after transfection of AKT1 and mTOR reporter plasmids plus miR-27a* in HNSCC cells demonstrates the 3'UTR of both signaling mediators have functional binding sites for miR-27a*, *p<0.001.

Given that miR-7 has known interactions with EGFR as both a tumor suppressor and oncogene [25, 29], but did not show an effect on cell viability in HNSCC, we postulated that miR-27a* targets additional genes in the EGFR signaling axis to reduce cell survival. Further *in silico* analysis identified AKT1 and mTOR as additional targets of miR-27a* (Fig. 3A). These findings were confirmed with analysis of protein expression in HNSCC cells transfected with miR-27a*, resulting in reduced protein levels of EGFR, AKT1 and mTOR compared to miR-control (Control). In contrast, miR-27a and −7 decreased EGFR expression without modulation of AKT1 or mTOR (Figs. 3B and 3C). Moreover, we confirmed the effect of miR-27a* on mRNA levels by qRT-PCR (Fig. S3A). Transfection of HNSCC cells with miR-27a*-IH slightly increased EGFR, AKT1 and mTOR expression as compared to miR-27a* and -Control (Fig. S3B). In HOK16B, EGFR expression was decreased by miR-27a* and slightly increased by miR-27a*-IH. Taken together, these results demonstrate post-transcriptional regulation of EGFR, AKT1, and mTOR by miR-27a*.

### Coordinated Downregulation of EGFR, AKT1 and mTOR is the Result of Independent, Direct Interactions with miR-27a*

To verify a direct interaction between miR-27a* and its target genes, we created reporter plasmids with inserted sequences from EGFR, AKT1 or mTOR downstream of a luciferase gene. E3908-pGL3 encompasses the 3'UTR, E2145-pGL3 spans a portion of the CDS, and E160-pGL3 contains a portion of the CDS upstream of E2145-pGL3 (Fig. 3D). Transfection of these EGFR reporter constructs and miR-27a* in HNSCC cells showed that the candidate binding site in E160-pGL3 was not functional (Fig. S3C). The remaining sites were deleted in their respective reporter constructs by site-directed mutagenesis to establish companion mutant vectors (MT). Transfection of E3908-pGL3 (WT) and miR-27a* decreased luciferase activity, but transfection of MT and miR-27a* did not, confirming that direct miR-27a* interaction with the 3'UTR contributes to decreased EGFR expression. Similarly, studies with E2145-pGL3 (WT) and (MT) demonstrated miR-27a* interactions with the 2 distal binding sites in the CDS (Fig. 3E). Reporter constructs for the 3'UTR of AKT1 and mTOR (Fig. S3D) demonstrated similar decreased transcriptional activity when introduced into HNSCC cells along with miR-27a* (Fig. S3E). Luciferase activity was not affected when the mutant (MT) reporter vectors for AKT1 and mTOR were used (Fig. 3F). Similarly, miR-27a*-IH did not affect luciferase activity with the E3908-pGL3 (WT), AKT1-pGL3, mTOR-pGL3 or MT counterparts (Fig. S3F). These results confirm a direct interaction between miR-27a* and its respective targets EGFR, AKT1, and mTOR.

### Overexpression of EGFR, AKT1 and mTOR Inhibits the Effect of miR-27a* on HNSCC Cell Viability

**Figure 4:**
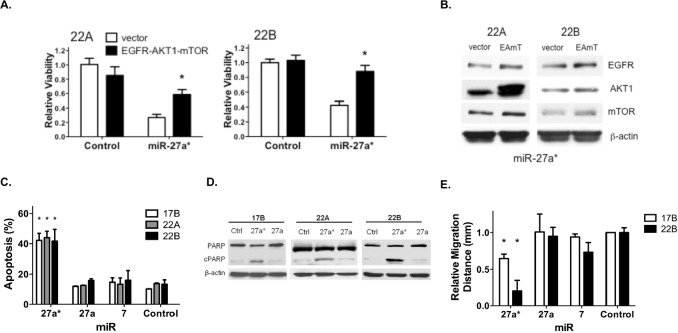
Overexpression of EGFR axis signaling components reverses the loss of HNSCC cell viability mediated by miR-27a*, which increases apoptosis and reduces migration (A) The effect of miR-27a* on cell viability is decreased in 22A and 22B cells by overexpression of EGFR, AKT1 and mTOR 48 hrs prior to miR-27a* expression as compared to control vector, *p<0.01; (B) Overexpression following transfection with EGFR, AKT1 and mTOR (EAmT) vectors was confirmed by immunoblot analysis. (C) Annexin V assay shows increased apoptosis in HNSCC cells after transfection with miR-27a*, *p<0.005; (D) Transfection with miR-27a* leads to increased PARP cleavage compared to miR-27a and -Control; (E) Wound healing assay demonstrates decreased migration distance after 24 hrs after transfection of miR-27a* compared to miR-Control, −27a, and −7, *p<0.001.

Although miR-27a* targets EGFR, AKT1 and mTOR directly and independently within the EGFR signaling pathway, the specific effect of those regulatory events on the overall decreased viability of HNSCC cells has not been established. EGFR, AKT1 and mTOR CDS expression vectors were transfected into HNSCC cells prior to the introduction of miR-27a*. Overexpression of EGFR, AKT1 and mTOR decreased the effect of miR-27a* expression on HNSCC cell viability as compared to control vector alone (Fig. 4A). However, expression of these EGFR signaling pathway components did not completely abrogate the effect of miR-27a* on HNSCC cell viability. We confirmed increased expression of EGFR, AKT1 and mTOR by immunoblotting analysis (Fig. 4B). Thus, these findings suggest that the effect of miR-27a* expression on HNSCC cell viability was related, at least in part, to the EGFR signaling pathway.

### Inhibition of the EGFR Signaling Axis by miR-27a* Increases Apoptosis and Decreases Cellular Migration in HNSCC

Having demonstrated coordinate downregulation of the EGFR signaling axis, we analyzed the functional effects of miR-27a* in HNSCC. Cell cycle analysis indicated a marked increase in the sub-G1 apoptotic fraction (Figs. S4B and S4C) and Annexin V staining showed a significant increase in both early and late apoptotic cells, following miR-27a* transfection but not transfection of miR-27a and −7 (Figs. 4C and S4A). PARP cleavage verified the increase in apoptosis following transfection of miR-27a* into HNSCC cells (Fig. 4D). A wound healing assay revealed decreased migration in two HNSCC cell lines after transfection of miR-27a* as compared to miR-27a, −7 (Fig. 4E). Combined with the viability assays, these results expand the tumor suppressive functions of miR-27a* to include apoptosis and impaired migration in HNSCC cells.

### *In vivo* Expression and Delivery of miR-27a* Reduces HNSCC Tumor Growth

**Figure 5:**
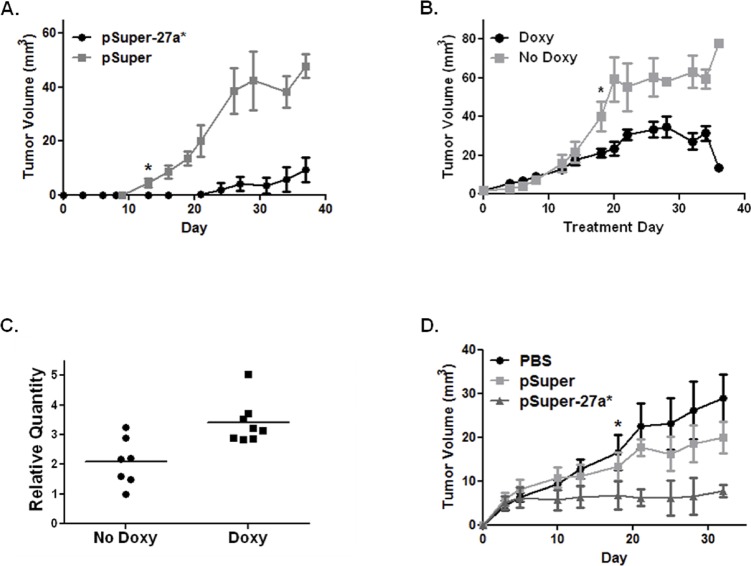
miR-27a* expression reduces tumor growth *in vivo* and direct intratumoral injection reduces tumor growth (A) Orthotopic xenografts of 22B cells expressing miR-27a* (pSuper-27a*) show reduced growth compared to control vector (pSuper); * at day 16 indicates point at which differences in tumor volume became statistically significant, p<0.05; (B) Tumor growth curve of 22B cells with inducible miR-27a* expression. One group of mice was treated with doxycycline (Doxy) to induce miR-27a* expression after tumors developed (day 0). Tumor volumes were significantly decreased in mice who received doxycycline; * marks day 18 when differences in tumor volumes became statistically significant, p<0.05; (C) Scatter plot depicting a statistically significant increase in miR-27a* RNA collected from murine tumor tissue in the doxycycline group compared to the non-doxycycline group, p<0.01; (D) Direct intratumoral injection significantly reduces growth of 17B tumors in mice treated with pSuper-27a* compared to control and pSuper; * marks day 18 when differences in tumor volumes became statistically significant (p<0.05) for pSuper-27a* compared to the control groups.

In order to assess the therapeutic potential of miR-27a* in a preclinical model, we created cell lines that stably express miR-27a*. 22B-pSuper-27a* constitutively expresses miR-27a* and decreases the expression of EGFR, AKT1, and mTOR. In a murine orthotopic xenograft model of oral cavity cancer, tumors from 22B-pSuper-27a* cells grew significantly slower than tumors from 22B-pSuper control cells (Fig. 5A). 22B-pSingle-27a* contains an inducible vector system resulting in miR-27a* expression when treated with doxycycline. As expected, the doxycycline-treated mice had significantly decreased tumor growth compared to the control group (Fig. 5B). Increased miR-27a* expression was confirmed by qRT-PCR in the tumors of the doxycycline-treated animals as compared to the control animals (Fig. 5C).

We also assessed the potential for exogenous delivery of miR-27a* to affect the growth of 17B cells *in vivo* by local delivery of miR-27a* using a liposomal vehicle. Tumors were established using 17B cells and then randomized into three groups: PBS, pSuper-27a*, and pSuper-empty. The mice treated with pSuper-27a* were found to have significantly reduced growth compared to the other treatment groups (Fig. 5D). These results suggest that miR-27a* alters HNSCC tumor growth *in vivo* and can serve as a potential therapeutic regimen in the treatment of patients with HNSCC.

## DISCUSSION

Our work identifies a miRNA* that coordinately regulates multiple components within a given signaling pathway. MiR-27a* simultaneously decreases expression of EGFR, AKT1, and mTOR leading to decreased solid tumor viability. Unlike miRNAs, which have evolved to regulate biologically related targets, targeted therapies often have non-specific effects on other cell targets, which may result in significant toxicities [30]. Similarly, current therapeutic approaches to HNSCC have to balance the increased efficacy of combined treatment modalities (e.g., radiation therapy and cisplatin) with the risk of increased side effects [31]. Thus, utilizing miR-27a* expression as a treatment option for HNSCC may be more effective and result in fewer unintended consequences.

MiR-27a* is noteworthy because it is one of the first complementary sequences with an identified functional effect in human cancers. This expands upon a previous working model in which miRNA* was thought to be a processing byproduct of the forward miRNA sequence [32-34]. Deep sequencing studies have demonstrated that miRNA* are abundant and conserved across multiple species leading to the speculation that they may have a significant regulatory role in cellular function [32, 35]. This theory is supported by our studies as miR-27a* transfection into HNSCC cells had significant functional effects while its complementary strand, miR-27a, did not. Additionally, our findings emphasize the need to clearly distinguish the effects of forward and star miRNA sequences.

MiR-27a was previously described as an oncogenic miRNA in pancreatic and bronchial cells [36, 37]; however, our results show that miR-27a is downregulated in HNSCC and its re-introduction into HNSCC cells does not have a pro-tumorigenic effect. This suggests differential expression of miR-27a target genes across multiple tumor types and divergent functional effects. Most importantly, miR-27a* demonstrated a tumor-suppressive function that miR-27a did not exhibit in HNSCC cells, which may potentially be the result of an alteration in the processing machinery. Differential expression of miRNA/miRNA* has been previously described not only across species but also within an individual due to alterations in the miRNA biogenesis machinery [38-40]. Significant work is still required to identify cellular factors that influence the fate of miRNA and miRNA* strands.

The interaction of miRNAs with the CDS in murine cell lines has been reported with the suggestion that this interaction may occur in human cells as well [41]. The interaction of miR-27a* with the CDS of the EGFR sequence, as observed in our work, appears to be the first example of this interaction in human cells. These findings and our characterization of multiple miRNA* binding sites within a single gene highlights the need for future studies to understand miRNA* processing and the precise nature of miRNA interactions with their target genes.

The use of RNA interference as a therapeutic modality in human disease is an attractive option because of the variable effects of small molecule inhibitors [42]. However, systemic delivery of small RNAs is met with major limitations, such as poor specificity and cellular uptake, degradation by nucleases, and rapid renal clearance [43]. Liposomal complexes have been found to improve cellular uptake of small RNAs in ovarian carcinoma and non-small cell lung cancer models [44, 45]. Specificity can be overcome in many head and neck tumors because direct access and visualization permits intratumoral injection of small RNAs as demonstrated in our orthotopic tongue model. Despite the current issues related to delivery, further investigation into the therapeutic potential of miR-27a* is warranted in not only HNSCC, but also in other readily accessible solid tumors.

In conclusion, we have identified miR-27a* as a regulator of multiple targets within the EGFR signaling axis. MiR-27a* expression significantly reduced the growth and viability of HNSCC in a preclinical model. Overexpression of EGFR signaling pathway components decreased the overall effect of miR-27a* on HNSCC cell viability, suggesting the possibility that other signaling pathways may also be affected by miR-27a*. Inhibition of miR-27a* did not significantly affect cell viability, but did result in a slight increase in targeted protein expression. These findings will drive future studies to examine other pathways regulated by miR-27a* and to understand potential loss-of-function effects related to miR-27a*. Nevertheless, our current studies suggest that miR-27a* is an attractive therapeutic option for the treatment of solid tumors. Finally, by targeting several components of the EGFR axis, miR-27a* may enhance the effect of existing treatment options and possibly provide therapeutic benefits to patients with EGFR-inhibitor resistant cancers.

## MATERIALS AND METHODS

### miRNA Predictions

miRGen, TargetScan and RNA22 were used to predict miRNA target sites in the mRNA sequences of EGFR, mTOR and AKT1.

### Cell Lines

All cell lines were cultured under standard conditions and verified by short tandem repeat profiling.

### qRT-PCR for miRNA and mRNA

RNA was extracted with Trizol (Invitrogen). cDNA was synthesized with either the Taqman miRNA Reverse Transcription Kit (Ambion) or with the SuperScript First-Strand Synthesis System (Invitrogen).

### Transfection with miRNA mimics, inhibitors and stem-loop structure

Cells were transfected with miRNA mimics (Ambion), miRNA inhibitors (Dharmacon) and the synthesized miR-27a step-loop structure (Dharmacon) using Lipofectamine 2000 (Invitrogen) per the manufacturer's instruction. Cells were harvested 24 (RNA) or 48 (protein) hrs later.

### Overexpression of EGFR, AKT1 and mTOR

Cells were co-transfected with Addgene expression vectors 11011, 9021, 26603 and pcDNA3.1 (Invitrogen). Cells were re-transfected with miRNA mimics 48 hrs after EGFR, AKT1 and mTOR overexpression.

### Viability assays

Cells were transfected with mimics and incubated for 72 hrs. MTT was then added and after 4 hrs, the supernatant was replaced with DMSO. OD values were measured at 570 nm. Alternatively, 1/10 volume of alamarBlue® (Invitrogen) was added and incubated at 37°C. Fluorescent measurements were taken at 530 nm and 590 nm.

### Apoptosis assays

HNSCC cells were analyzed 72 hrs after miRNA transfection. Samples were stained with propidium iodide and analyzed by flow cytometry. Alternatively, the PE Annexin V Apoptosis Detection Kit I (BD Biosciences) was used.

### Immunoblot analysis

Proteins were separated using SDS-PAGE, transferred to nitrocellulose membranes, then incubated overnight with primary antibodies for EGFR (BD Bioscience), mTOR (Upstate), AKT1 (Cell Signaling) or PARP (Cell Signaling). Densitometry was performed using Multi Gauge V3.0 software.

### Luciferase reporter construction and assay

Regions of EGFR, AKT1 and mTOR were cloned into pCRII-TOPO and excised by *XbaI/SpeI* before splicing into pGL3-control. MT reporters were generated using the Change-it Multiple Mutation Site Directed Mutagenesis Kit (USB) according to the manufacturer's protocol. Primer sequences are presented in Supplementary Tables 1 and 2.

Cells were co-transfected with 100 ng of reporter constructs and 5 ng of *Renilla*. MiRNA mimics were transfected with Lipofectamine 2000 24 hrs later. Lysates were assayed with the Dual-Glo Luciferase Assay System (Promega).

### Establishment of miR-27a* expressing cell lines

The mature miR-27a* sequence was synthesized (Sigma Aldrich) and inserted into pSuper (Oligoengine) and pSingle (Clontech). Plasmids were transfected into 22B cells using Lipofectamine 2000. Clones were selected at 300 μg/ml G418 for 2 weeks.

### Orthotopic mouse models

Mice were injected with 1×10^6^ 22B-pSuper-27a*, 22B-pSuper and 22B-pSingle-27a* cells. For the 22B-pSingle-27a* experiment, the treatment group received 2 mg/ml doxycycline in water. To assess the delivery of miR-27a* *in vivo*, 17B oral tumors were directly injected with PBS, pSuper-empty, or pSuper-27a*, all mixed with liposome at a ratio of 5:1 [46, 47]. Tumors were measured twice a week.

### Statistics

All values are expressed as means ± standard error. Correlations between parameters and endpoints were assessed using a two-tailed Student's *t* test. A value of p<0.05 was considered statistically significant.

## Supplementary Figures, Tables and Methods






