# Supplementary material for: Coordinated Targeting of the EGFR Signaling Axis by MicroRNA-27a*
Source: Oncotarget. 2013 Aug 6;4(9):1388–98. doi: 10.18632/oncotarget.1239 (PMC3824521; doi:10.18632/oncotarget.1239)
Supplement: Supplementary file 1 [file oncotarget-04-1388-s001.pdf]

Coordinated Targeting of the EGFR Signaling Axis by MicroRNA-27a* - Wu et al

SUPPLEMENTARY FIGURES

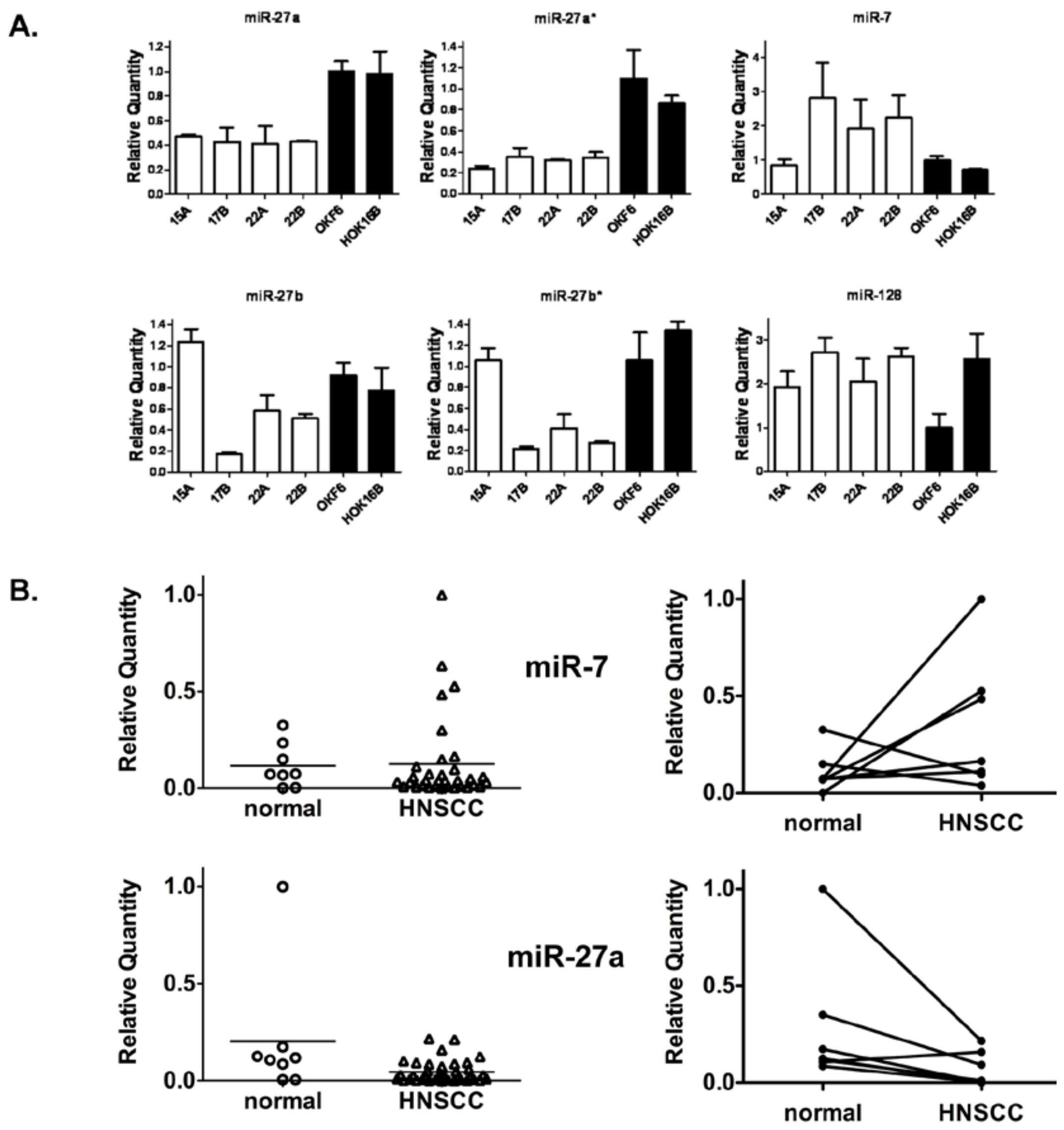

Supplementary Figure 1: miR-27a* has decreased expression compared to other EGFR-targeting miRNAs in HNSCC. (A) Expression of mature miR-27a, -27a*, -27b, -27b*, -7 and -128 by qRT-PCR in four HNSCC cell lines and transformed oral keratinocytes (OKF-6 and HOK16B). Values normalized to OKF-6; (B) Analysis of miR-7 and miR-27a in human HNSCC and normal mucosal specimens by qRT-PCR did not reveal a statistically significant difference in miR-7 expression levels in HNSCC as compared to normal tissue samples in the overall cohort or matched normal/HNSCC tissue pairs. MiR-27a was significantly decreased in HNSCC as compared to normal tissue samples in the overall cohort ($p<0.05$), but not in the matched normal/HNSCC tissue pairs.

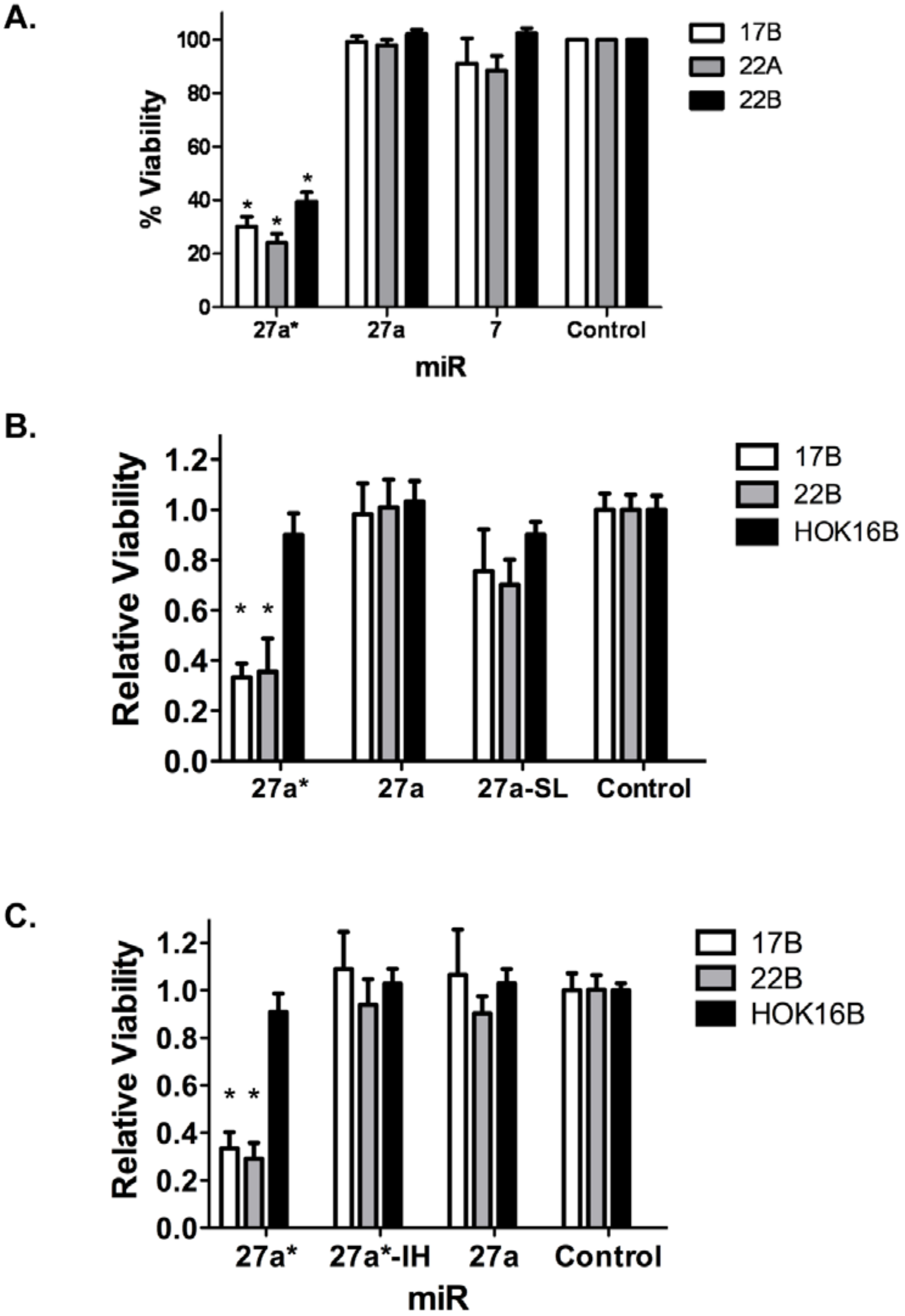

Supplementary Figure 2: miR-27a* transfection reduces HNSCC cell viability. (A) Cell viability was decreased in three HNSCC cell lines after transfection with miR-27a* using alamarBlue® assay, *p<0.01; (B) Cell viability was decreased in HNSCC cells (17B and 22B) to a greater degree following transfection with miR-27a* than the miR-27a stem-loop structure (miR-27a-SL) using MTT assay, *p<0.01. Cell viability of transformed oral keratinocytes (HOK16B) was not significantly affected by miR-27a*, miR-27a or miR-27a-SL; (C) Cell viability was decreased in HNSCC cells, but not transformed oral keratinocytes (HOK16B) by miR-27a* using MTT assay, *p<0.01. Transfection with an antagomir, miR-27a*-inhibitor (miR-27a*-IH), did not affect cell viability in HNSCC or HOK16B cells.

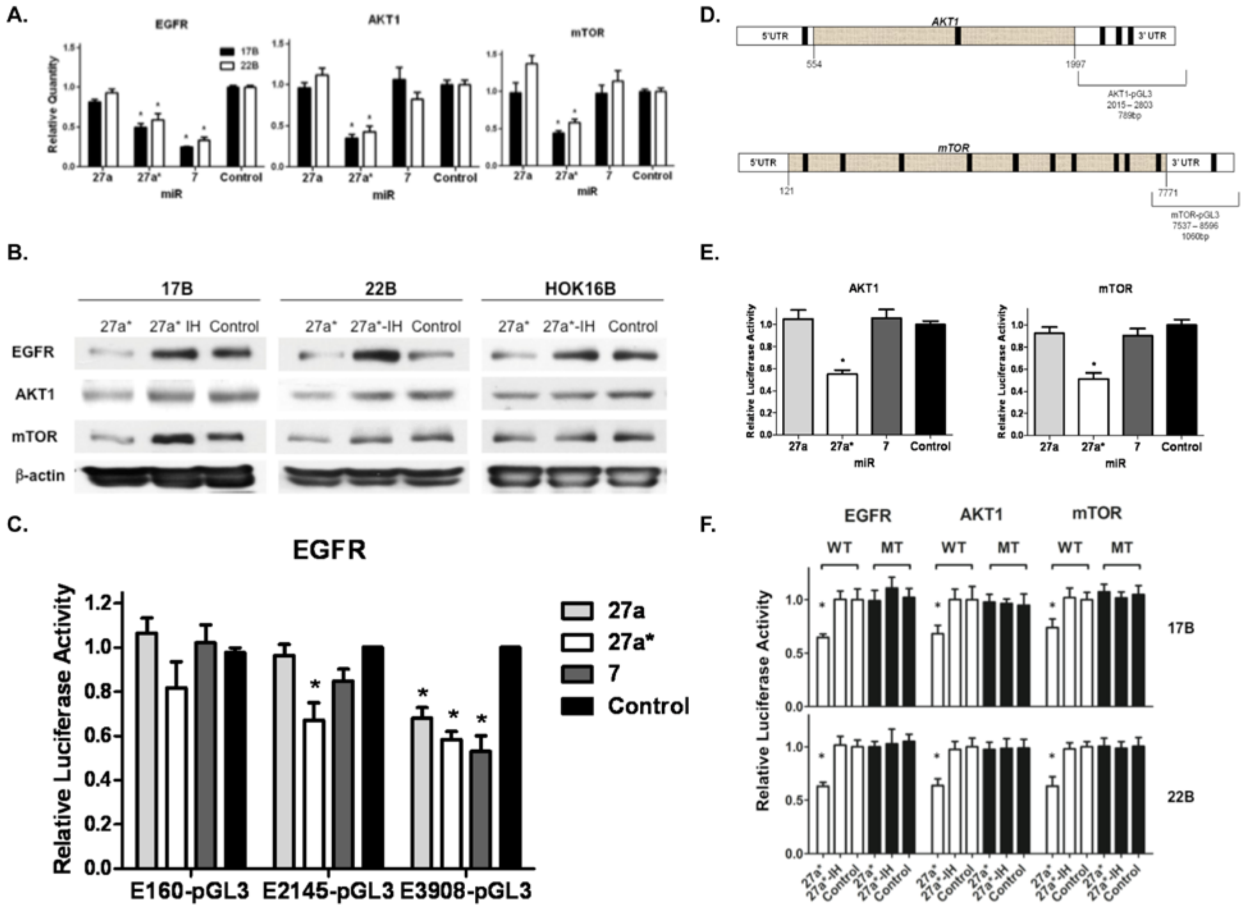

Supplementary Figure 3: miR-27a* causes coordinated mRNA degradation through direct interaction with EGFR, AKT1 and mTOR, while inhibition of miR-27a* does not affect HNSCC cell viability. (A) Relative values of EGFR, AKT1, and mTOR mRNA after transfection of miR mimics in HNSCC cells using qRT-PCR. Note, miR-27a* decreases all three mediators in the signaling axis. Values are normalized to miR-Control, *p<0.001; (B) Immunoblot shows decreased EGFR, AKT1 and mTOR expression in HNSCC cells with miR-27a* expression, but increased expression with miR-27a*-IH. EGFR expression is decreased with miR-27a* expression and increased with miR-27a*-IH transfection in HOK16B cells; (C) Relative luciferase activity normalized to control in 17B cells after co-transfection of EGFR reporter vectors with miR-27a*, -27a, -7 or -Control. MiR-27a* shows significant decrease at sites within the CDS (segment 2145) and 3'UTR (segment 3908). MiR-27a and -7 also show binding, as expected, in the 3'UTR but not the CDS, *p<0.05; (D) Map of reporter plasmid constructs depicting the 3'UTR sequences of AKT1 and mTOR placed downstream of the luciferase gene in pGL3; (E) Relative luciferase values normalized to control after co-transfection of either the AKT1 3'UTR reporter or mTOR 3'UTR reporter with miR-27a*, -27a, -7, or -Control in 17B cells. Note the significant decrease in luciferase seen with miR-27a* that is not observed in either miR-27a or -7, *p<0.001; (F) Luciferase assay activity after transfection with EGFR, AKT1 or mTOR reporter plasmids and miR-27a* in HNSCC cells demonstrates direct interaction of miR-27a* with the 3'UTR of the respective genes. No alteration in luciferase activity is detected with transfection of miR-27a*-IH in these cells, *p<0.05.

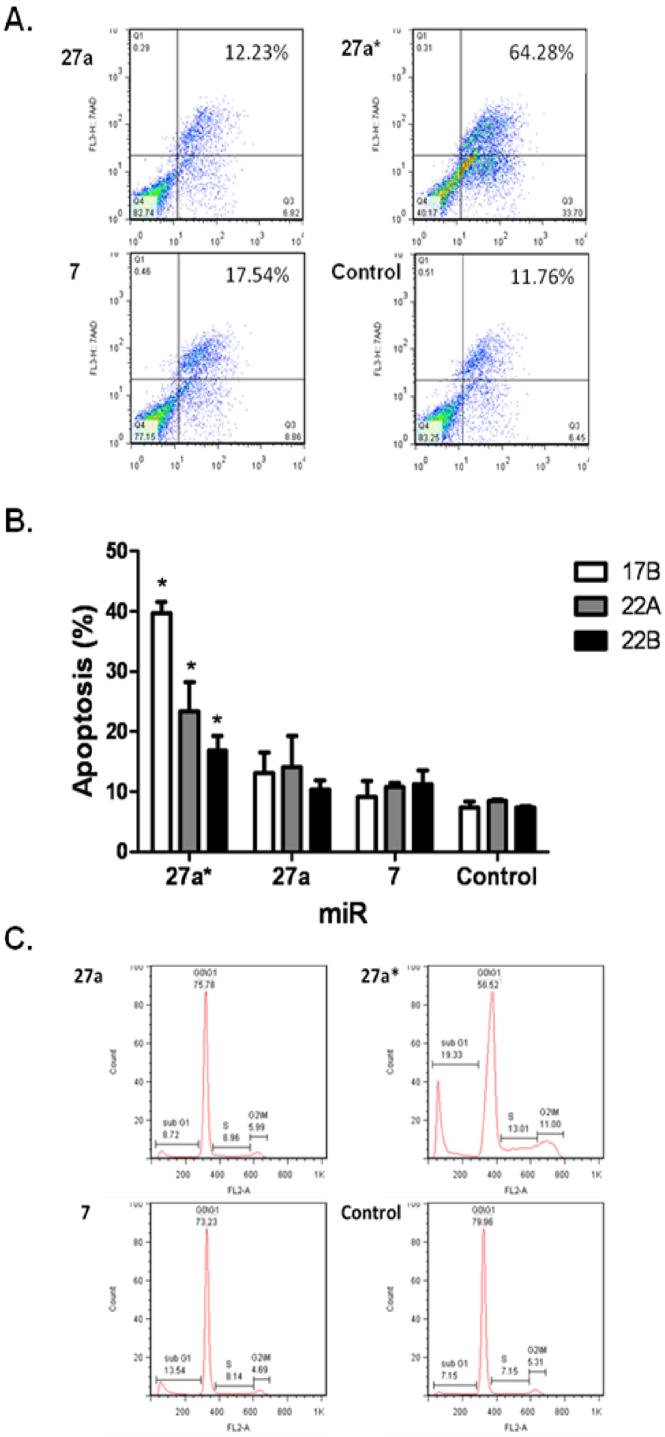

Supplementary Figure 4: miR-27a* increases apoptosis in HNSCC. (A) Annexin V assay shows 22B cells transfected with miR-27a* have higher apoptotic fraction (upper right quadrant) compared to miR-27a, -7 and –Control; (B) Cell cycle analysis shows 3 HNSCC cell lines with increased apoptosis after transfection with miR-27a* compared to miR-27a, -7, -Control, *p<0.05; (C) Corresponding flow cytometry analysis after PI staining during cell cycle analysis shows 22B cells transfected with miR-27a* in an increased sub-G1 fraction compared to miR-27a, -7, -Control.
