# Supplementary material for: Coordinated Targeting of the EGFR Signaling Axis by MicroRNA-27a*
Source: Oncotarget. 2013 Aug 6;4(9):1388–98. doi: 10.18632/oncotarget.1239 (PMC3824521; doi:10.18632/oncotarget.1239)
Supplement: Supplementary file 2 [file oncotarget-04-1388-s002.pdf]

Coordinated Targeting of the EGFR Signaling Axis by MicroRNA-27a* - Wu et al

Supplementary Table 1. Primer sequences used to create wild-type luciferase reporter plasmids.

Plasmid	Direction	Primer
E160-pGL3 WT	F	5’-CGCCAACGCCACAACCAC-3’
	R	5’-CGCCGTCTTCCTCCATCT-3’
E2145-pGL3 WT	F	5’-GCTCTAGATGAAGGCTGTCCAACGAA-3’
	R	5’-GCTCTAGAATCAGGGCACGGTAGAAG-3’
E3908-pGL3 WT	F	5’-GCTCTAGATCCAGACTCTTTCGATACC-3’
	R	5’-GCTCTAGACAAATACTTTCTTAACAATGCT-3
AKT1-pGL3 WT	F	5'-CTGGACGATAGCTTGGAGG-3'
	R	5'-CGACAGCGGAAAGGTTAA-3'
mTOR-pGL3 WT	F	5'-GACCACAGTGCCAGAATC-3'
	R	5'-ATATGTACCAGACCTTCCCT-3'

Supplementary Table 2. Primer sequences used to create mutant luciferase reporter plasmids.

Plasmid	Deletion		Primer
E160-pGL3 MT	nt.355-360	F	5' CAAGGCACGAGTAACACGCAGTTGGGCACT 3'
		R	5' AGTGCCCAACTGCGTGTTACTCGTGCCTTG 3'
E2145-pGL3 MT	nt.2226-2231	F	5' CTTGCTGCTGGTGGTGGGGATCGGC 3'
		R	5' GCCGATCCCCACCACCAGCAGCAAG 3'
	nt.3216-3222	F	5' ATGAAAGAATGCATTTGCCACAGACTCCAACTTCTACC 3'
		R	5' GGTAGAAGTTGGAGTCTGTGGCAAATGCATTCTTTCAT 3'
E3908-pGL3 MT	nt.4363-4368	F	5' CCAGAGGATGCTTGATTCCAGTGCTTCAAGGCTT 3'
		R	5' AAGCCTTGAAGCACTGGAATCAAGCATCCTCTGG 3'
	nt.5099-5104	F	5' TCTATTCAAGCACTTACGGCCACAACAGGGCATT 3'
		R	5' AATGCCCTGTTGTGGCCGTAAGTGCTTGAATAGA 3'
	nt.5354-5359	F	5' GTAACCTGACTGGTTAACAGCTTGTAAACAGTGTTTTAAACTC 3'
		R	5' GAGTTTAAAACACTGTTTACAAGCTGTTAACCAGTCAGGTTAC 3'
	nt.5525-5530	F	5' TTTGACTCCCAGATCAGTCACTACAGCATTGTTAAGAAAG 3'
		R	5' CTTTCTTAACAATGCTGTAGTGACTGATCTGGGAGTCAAA 3'
AKT1-pGL3 MT	nt.2242-2247	F	5' CCGGGTGTGGCCTCCAGAACAATCCGAT 3'
		R	5' ATCGGATTGTTCTGGAGGCCACACCCGG 3'
	nt.2543-2549	F	5' ACGTCATCGGAGGCCTGGGATGGGAC 3'
		R	5' GTCCCATCCCAGGCCTCCGATGACGT 3'
	nt.2681-2687	F	5' GGTTTTTAATCTTTGTGACAGGACCCCCTTCCCCTT 3'
		R	5' AAGGGGAAGGGGGTCCTGTCACAAAGATTAAAAACC 3'
mTOR-pGL3 MT	nt.7695-7701	F	5' GGATGTTCCAACGCAAGGCTCATCAAACAAGCGA 3'
		R	5' TCGCTTGTTTGATGAGCCTTGCGTTGGAACATCC 3'
	nt.8490-8495	F	5' GGCCTTCTGCAGCAGGGGATGTTTTCAGTG 3'
		R	5' CACTGAAAACATCCCCTGCTGCAGAAGGCC 3'
