# Supplementary material for: Coordinated Targeting of the EGFR Signaling Axis by MicroRNA-27a*
Source: Oncotarget. 2013 Aug 6;4(9):1388–98. doi: 10.18632/oncotarget.1239 (PMC3824521; doi:10.18632/oncotarget.1239)
Supplement: Supplementary file 3 [file oncotarget-04-1388-s003.pdf]

Coordinated Targeting of the EGFR Signaling Axis by MicroRNA-27a* - Wu et al

SUPPLEMENTARY METHODS

MicroRNA computational predictions

The web-based programs miRGen (http://www.diana.pcbi.upenn.edu/miRGen.html) and TargetScan (http://www.targetscan.org/) were used to investigate the 3'UTR mRNA sequences of human EGFR, mTOR and AKT1 for putative binding sites of miRNAs. RNA22 (cbcsrv.watson.ibm.com/rna22.html) was used to identify miRNA target sites in the whole mRNA sequence of EGFR, mTOR and AKT1, their corresponding RNA/RNA complexes and folding energy.

Cell lines and maintenance

HNSCC cell lines UMSCC-10A, -10B, -11A, -11B, -15A, -15B, -17A, -17B, -22A, -22B were cultured at 37°C and 5% CO_2 in Dulbecco's modified Eagle's medium (DMEM, Mediatech, MT10013CV) containing 10% heat-inactivated FBS. Immortalized human oral keratinocyte cell lines HOK16B and OKF6 were cultured in Keratinocyte-Serum-Free Media (Invitrogen, SKU# 37010-022) containing 5% FBS, Bovine Pituitary Extract (BPE, 25 µg/ml), and Epidermal Growth Factor (EGF, 0.2 ng/ml). The breast cancer cell line MDA-468 was cultured in DMEM/F12 (Mediatech, Inc., Herndon, VA, MT10090CV) supplemented with 10% FBS. The endometrial carcinoma cell line, HEC1A, was cultured in McCoy's 5A media supplemented with 10% FBS and penicillin/streptomycin (pen/strep, Sigma). PC3, prostate adenocarcinoma, was cultured in RPMI with 10% FBS and pen/strep. The pancreatic adenocarcinoma cell line, MIA PaCa, was cultured in DMEM with 10% FBS and pen/strep. All cell line identities were verified using short tandem repeat profiling.

qRT-PCR to quantify mature miRNA

Total RNA was extracted with Trizol (Invitrogen). RNA concentration was determined using a ND-1000 Spectrophotometer (NanoDrop Technologies). For mature miRNA expression analysis, cDNA was synthesized using Taqman MicroRNA Reverse Transcription Kit (Applied Biosystems, PN 4366596) with 100 ng total RNA (100 ng/µl) and 1 µl of specific primers for miR-27a, -27a*, 27b, -27b*, -7, -128 supplied with the miR Taqman MicroRNA Assay (Applied Biosystems) and performed according to the manufacturer's instructions. Quantitative real-time PCR (qRT-PCR) analysis was performed using the 7900HT Real-Time PCR System (Applied Biosystems). The 15 µl PCR reaction included 5 µl cDNA, 0.75 µl Taqman miRNA assay probe (supplied with miR Taqman MicroRNA Assay), 7.5 µl Taqman 2X Universal PCR master mix (Applied Biosystems, 4304437), and 1.75 µl water. The reactions were incubated in a 96-well plate at 95°C for 10 min, followed by 40 cycles of 95°C for 15 sec, and 60°C for 1 min. All reactions were done in triplicate. RNU6B (Applied Biosystems) was used as endogenous control. To assess miR-27a* levels in human tissue specimens, we examined a cohort of 47 specimens from patients presenting with oral tongue squamous cell carcinoma, representing 37 tumors and 10 normal biopsies. Tumor and normal specimen pairs were available from seven patients. Tissue specimens were microdissected from frozen tissue specimens for preparation of RNA samples and were used to evaluate the expression of miR-7, miR-27a and miR-27a*. Access to

the human tissue specimens was approved by the University of Texas MD Anderson Cancer Center Institutional Review Board.

qRT-PCR to quantify EGFR, AKT1 and mTOR mRNA

Total RNA was extracted with Trizol (Invitrogen). DNase I, Amplification Grade (Invitrogen) and SuperScript First-Strand Synthesis System for RT-PCR (Invitrogen) were used for cDNA preparation. Primers and probes were ordered from IDT Inc. The reactions were incubated in a 96-well plate at 95°C for 12 min, followed by 40 cycles of 95°C for 30 sec, 60°C for 1 min, and 72°C for 45 sec as previously described [48]. All reactions were done in triplicate.

Cell line transfection with miRNA mimics

Cells were seeded 24 hours prior to transfection in 6-well plates or 6 cm dishes and transfected using Lipofectamine 2000 (Invitrogen) with miRNA mimics at a final concentration of 50 pM. Synthetic miRNA mimic molecules corresponding to human miR-27a, -27a*, -7 and a negative control (-Control) were purchased from Ambion. Cells were harvested at 24 hours (for RNA extraction) or 48 hours (for protein extraction) after transfection.

Overexpression of EGFR, AKT1 and mTOR

Cells were co-transfected with CDS expression vectors: pBABE-EGFR WT (Addgene plasmid 11011 created by Dr. M. Meyerson; [49]); 1477 pcDNA3-Flag HA Akt1 (Addgene plasmid 9021 created by Dr. W.R. Sellers; [50]); and pcDNA3-Flag mTOR WT (Addgene plasmid 26603 created by Dr. J. Chen; [51]). pcDNA3.1 empty plasmid (Invitrogen) was used as vector control. Cells were re-transfected with miRNA mimics 48 hrs after EGFR, AKT1 and mTOR overexpression. Cell were harvested 48 hrs after miRNA transfection for protein analysis.

AlamarBlue® assay

Seventy-two hours after miRNA mimics were transfected into cells, one-tenth volume of alamarBlue® (Invitrogen) was added and plates were incubated at 37°C until a color change was noted. Fluorescence was measured with excitation at 530 nm and emission at 590 nm on a Synergy HT Multi-Mode Microplate Reader (Bio-Tek). All reactions were done in triplicate.

MTT assay

After transfection with miRNA mimics and seventy-two hour incubation, MTT (3-(4, 5-dimethylthiazol-2-yl)-2, 5-diphenyl tetrazolium bromide) was added into the wells at a final concentration of 1 mg/ml. After incubation at 37°C for 4 hours, the supernatant was discarded and dimethyl sulfoxide (DMSO) was added to dissolve the formazan. Optical density (OD) values were measured at a wavelength of 570 nm with a Synergy HT Multi-Mode Microplate Reader (Bio-Tek). All reactions were done in triplicate.

Cell cycle analysis

Cells were seeded in 6-well plates and analyzed 3 days after miR-27a, -27a*, -7 or -Control transfection. Following trypsinization, cells were permeabilized by phosphate-citrate (PC) buffer, stained with propidium iodide (PI), and analyzed on a BD FACSCalibur Flow Cytometer (BD Biosciences). Cell cycle analysis was performed using Flowjo Flow Cytometry Analysis Software.

Annexin V staining

Apoptosis was measured by flow cytometry to quantify the levels of detectable phosphatidylserine (PS) on the outer membrane of apoptotic cells bound to Annexin V. Cells were seeded in 6-well plates and analyzed 72 hrs after miR-27a, -27a*, -7 or -Control transfection. Following trypsinization, cells were washed twice with cold PBS and resuspended in binding buffer. The cell suspensions were then incubated with 5 µL of Annexin V for 15 min. Additional binding buffer and 20 µL of 7-aminoactinomycin (AAD) were added before the population of Annexin V/7-AAD positive cells were evaluated by flow cytometry. Annexin V assay was performed using the PE Annexin V Apoptosis Detection Kit I (BD Biosciences).

Western blot analysis

Cells were plated on 6 cm dishes. Subconfluent cells were washed in ice-cold PBS and harvested in lysis buffer (10 mM Tris-HCl, pH 7.6, 5 mM EDTA, 50 mM NaCl, 30 mM $Na_4P_2O_7$, 50 mM NaF, 1 mM Na_3VO_4, 1% Triton X-100, Complete Mini tablet, 0.8 mM DTT and 1.6 mM PMSF). The cells were sonicated and centrifuged, and the protein concentrations of the cell lysates were determined using the Bio-Rad Protein Assay (Bio-Rad). Protein lysates (30 µg per lane) were separated by sodium dodecyl sulfate-polyacrylamide gel electrophoresis (SDS-PAGE) and transferred to nitrocellulose membranes. Membranes were incubated in 5% milk for 2 hrs at room temperature, and then incubated with primary antibodies for EGFR (BD Bioscience), mTOR (Upstate), AKT1 (Cell Signaling) or poly (ADP-ribose) polymerase (PARP; Cell Signaling) at 1:1000 dilution overnight at 4°C. Horseradish peroxidase (HRP) conjugated goat anti-mouse IgG antibody (Bio-Rad) or HRP-conjugated goat anti-rabbit IgG antibody (Bio-Rad) was used at 1:3000 dilution for 2 hrs at room temperature. Membranes were washed with TBST and proteins were detected with the Western Blotting Luminol Reagent (Santa Cruz Biotechnology). β-actin was used as an endogenous control.

Densitometry was performed using the Multi Gauge V3.0 software. Pixel intensity was measured in identical rectangular areas around each band on scanned images of immunoblots, and a background value in an equal rectangular area separate from the bands was subtracted to obtain the mean pixel intensity/mm^2. Each sample was normalized to β-actin.

Luciferase reporter construction

Wild-type reporter plasmids for EGFR, AKT1 and mTOR were generated by insertion of 3 regions of EGFR (GenBank accession number NM_005228), 3'UTR of AKT1 (GenBank accession number NM_005163) and 3'UTR of mTOR (GenBank accession number NM_004958) into the *XbaI* restriction site 3' to luciferase in the pGL3-control plasmid and named E160-pGL3, E2145-pGL3, E3908-pGL3, AKT1-pGL3, and mTOR-pGL3. The three regions of EGFR, E160 (nt. 160-1213), E2145 (nt. 2145-3251) and E3908 (nt. 3908-5556) were amplified from human genomic DNA using synthesized primers (Supplementary Table 1). The amplified DNA sequences were cloned into the pCRII-TOPO plasmid and excised by *XbaI/SpeI* restriction enzyme before splicing into the pGL3-control vector. Accuracy of the amplified DNA sequences was verified by DNA sequencing.

Mutant (MT) reporters were generated by deletion of the predicted miR-27a* seed binding site in wild-type reporter plasmids using the Change-it Multiple Mutation Site Directed Mutagenesis

Kit (USB) according to the manufacturer's protocol. The primers used for the generation of mutant constructs are listed in Supplementary Table 2.

Luciferase assay

Cells were seeded in 24-well plates and co-transfected using Lipofectamine 2000 (Invitrogen) with 100 ng of firefly luciferase reporter constructs and 5 ng of β-actin promoter *Renilla* luciferase reporter as control. MiRNA mimics were transfected with Lipofectamine 2000 24 hrs later. Lysates were assayed for firefly and *Renilla* luciferase activities 48 hrs after the transfection of miRNA mimics using the Dual-Glo Luciferase Assay System (Promega) and a Sirius Single Tube Luminometer (Berthold Detection System). Expression values were normalized to *Renilla* luciferase.

Construction of pSuper and pSingle plasmids

The mature miR-27a* sequence was artificially synthesized (Sigma Aldrich) and inserted between *BglII* and *HindIII* restriction sites of the pSuper vector (Oligoengine). Plasmids were transfected into 22B cells using Lipofectamine 2000 and grown in standard conditions. Clones were selected in the presence of G418 (Sigma) at a dose of 300 µg/ml. Colonies were allowed to expand and individually selected. The clones were tested for miR-27a* expression, gene target knockdown, and decreased proliferation as described above.

For the inducible pSingle vector (Clontech), the miR-27a* sequence was artificially synthesized and inserted between the *XhoI* and *HindIII* restriction sites . The constructs were transfected into 22B cells and selected with G418 for 2 weeks. Individual colonies were selected and expanded in separate plates. The clones were tested with and without 1 µg/µl doxycycline (Sigma Aldrich) treatment for 72 hrs. Cell lysates and RNA were collected and tested for gene target knockdown and miR-27a* expression. Viability after exposure to doxycycline was assessed using the MTT assay.

Animals and maintenance

Male athymic nude mice, age 8 to 12 weeks, were purchased from the animal production area of the NCI-Frederick Cancer Research and Development Center (Frederick, MD). Rodent housing facilities at The University of Texas MD Anderson Cancer Center were approved by the American Association for Accreditation of Laboratory Animal Care in accordance with current regulations and standards of the US Department of Agriculture, the U.S. Department of Health and Human Services, and the NIH. Procedures and handling of mice were in accordance with the Animal Care and Use Guidelines of the University of Texas MD Anderson Cancer Center (Houston, TX) under a specified protocol approved by the Institutional Animal Care Use Committee.

Establishment of orthotopic nude mouse models using plasmids expressing miR-27a*

For the constitutive miR-27a* expression murine model, the oral tongues of nude mice were directly injected with $1x10^6$ 22B-pSuper-27a* and 22B-pSuper cells. Tumor growth was measured using calipers and volumes were calculated as $\pi lwh/6$. Values for each group were averaged and plotted as a function of days after tumor injection.

For the inducible miR-27a* expression murine model, 22B-pSingle-27a* cells were injected into the oral tongues as described above. Once tumors were visible (3-4 days after injection), mice were randomized into two groups, doxycycline (Doxy) treatment group and no-treatment. The doxycycline group received 2 mg/ml doxycycline dissolved in water with 5 mM saccharin (Sigma Aldrich). This mixture was refreshed every two days. The no-treatment group received 5 mM saccharin water without doxycycline. Tumor growth was measured as described above and plotted as a function of treatment day.

To assess the delivery of miR-27a* *in vivo,* the HNSCC cell line UMSCC-17B was implanted into the oral tongues of athymic nude mice. Mice were randomized into three different groups and tumors were directly injected with either PBS, pSuper-empty, and pSuper-27a*. The plasmids and PBS were mixed with liposome (Lipofectamine, Invitrogen, Inc.) at a plasmid:liposome ratio of 5:1. The total injection volume was 10 µl and mice were treated 3 days a week. Tumors were measured twice a week and volumes were calculated as above.

<u>Evaluation of miR-27a* in human tumor specimens</u>

We examined a cohort of patients treated with surgery at The University of Texas MD Anderson Cancer Center with oral tongue squamous cell carcinoma. The forty-seven available frozen and formalin-fixed paraffin-embedded (FFPE) specimens represented 37 tumors and 10 normal adjacent mucosal biopsies. In seven patients, paired tumor and normal tissue specimens were available. Tumors were microdissected from FFPE slides for preparation of RNA samples. RNA was extracted using Trizol reagent. qRT-PCR was performed as described above.

<u>Statistics</u>

All values are expressed as means±standard error. Correlations between parameters and endpoints were assessed using a two-tailed Student's *t* test. A value of $p<0.05$ was considered statistically significant.
